# Taxonomic review of *Saguinus mystax* (Spix, 1823) (Primates, Callitrichidae), and description of a new species

**DOI:** 10.7717/peerj.14526

**Published:** 2023-01-11

**Authors:** Gerson Paulino Lopes, Fábio Rohe, Fabrício Bertuol, Erico Polo, Ivan Junqueira Lima, João Valsecchi, Tamily Carvalho Melo Santos, Stephen D. Nash, Maria Nazareth Ferreira da Silva, Jean P. Boubli, Izeni Pires Farias, Tomas Hrbek

**Affiliations:** 1 Programa em Pós-Graduação em Zoologia, Universidade Federal do Amazonas, Manaus, Amazonas, Brazil; 2 Grupo de Pesquisa em Ecologia e Conservação de Primatas, Instituto de Desenvolvimento Sustentável Mamirauá, Tefé, Amazonas, Brazil; 3 Laboratório de Evolução e Genética Animal/Departamento de Genética/Instituto de Ciências Biológicas, Universidade Federal do Amazonas, Manaus, Amazonas, Brazil; 4 Grupo de Pesquisa em Ecologia de Vertebrados Terrestres, Instituto de Desenvolvimento Sustentável Mamirauá, Tefé, Amazonas, Brazil; 5 Programa de Pós-Graduação em Genética, Conservação e Biologia Evolutiva, Instituto Nacional de Pesquisas da Amazônia, Manaus, Amazonas, Brazil; 6 Programa de Pós-Graduação em Ecologia Aplicada, Universidade Federal de Lavras, Lavras, Minas Gerais, Brazil; 7 Rede de Pesquisa em Diversidade, Conservação e Uso da Fauna da Amazônia, Manaus, Amazonas, Brazil; 8 Comunidad de Manejo de Fauna Silvestre en América Latina, Iquitos, Peru; 9 Department of Anatomical Sciences/Health Sciences Center, Stony Brook University, New York, United States of America; 10 Instituto Nacional de Pesquisas da Amazônia, Manaus, Brazil; 11 School of Science, Engineering and the Environment, University of Salford, Salford, United Kingdom; 12 Department of Biology, Trinity University, San Antonio, Texas, United States

**Keywords:** Platyrrhini, Phylogeny, New world monkeys, New species, Cryptic, Amazonian tamarin, Phylogenomic, Geographic distribution, Taxonomy, Species

## Abstract

Although the Amazon has the greatest diversity of primates, there are still taxonomic uncertainties for many taxa, such as the species of the *Saguinus mystax* group. The most geographically broadly distributed and phenotypically diverse species in this group is *S*. *mystax*, and its phenotypic diversity has been recognized as three subspecies—*S*. *mystax mystax*, *S*. *mystax pileatus* and *S*. *mystax pluto*—with non-overlapping geographic distributions. In this sense, we carried out an extensive field survey in their distribution areas and used a framework of taxonomic hypothesis testing of genomic data combined with an integrative taxonomic decision-making framework to carry out a taxonomic revision of *S. mystax*. Our tests supported the existence of three lineages/species. The first species corresponds to *Saguinus mystax mystax* from the left bank of the Juruá River, which was raised to the species level, and we also discovered and described animals from the Juruá–Tefé interfluve previously attributed to *S*. *mystax mystax* as a new species. The subspecies *S*. *m*. *pileatus* and *S*. *m*. *pluto* are recognized as a single species, under a new nomenclatural combination. However, given their phenotypic distinction and allopatric distribution, they potentially are a manifestation of an early stage of speciation, and therefore we maintain their subspecific designations.

## Introduction

Marmosets and tamarins—species of the family Callitrichidae (Rylands et al., 2000, 2016)—are the most morphologically distinct and smallest of Neotropical primates (Hershkovitz, 1977). Marmosets and tamarins are currently classified in eight genera: *Leontopithecus* Lesson, 1840, *Callimico* Miranda-Ribeiro, 1911, *Saguinus* Hoffmannsegg, 1807, *Cebuella* Gray, 1866, *Callithrix* Erxleben, 1777, *Leontocebus* Wagner, 1839, *Mico* Lesson, 1840, and *Callibella* van Roosmalen & van Roosmalen, 2003 (Rylands et al., 2000; van Roosmalen & van Roosmalen, 2003; Buckner et al., 2015; Rylands et al., 2016; Silva et al., 2018).

**Figure 1:**
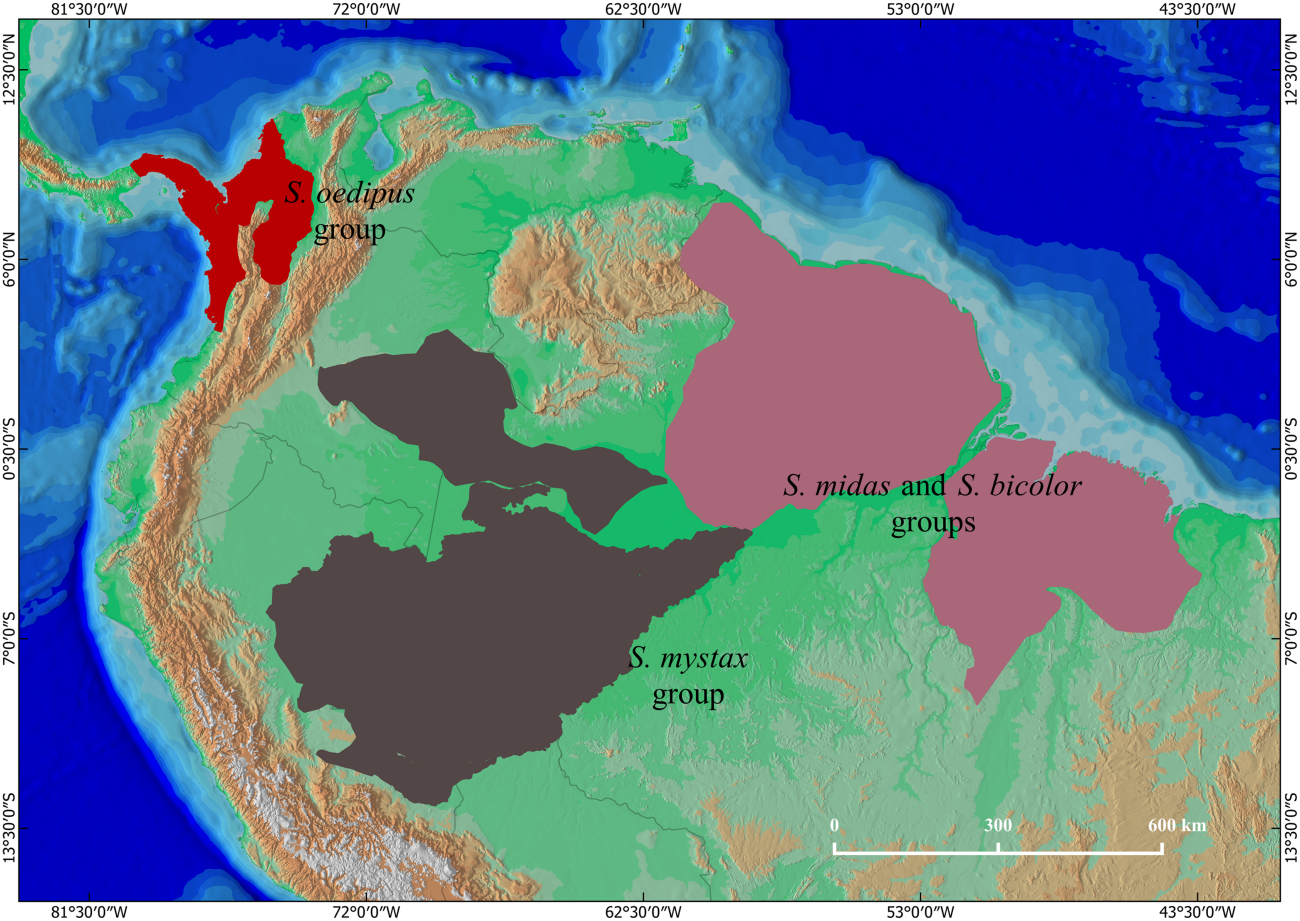
The geographical distributions of S*aguinus*: the *mystax* group (brown), the *oedipus* group (red), and the *bicolor* and *midas* groups (purple).

Tamarins of the genus *Saguinus* are distributed in forests of South and Central America (Hershkovitz, 1977; Fig. 1). Species of the genus *Saguinus* are currently organized into four species groups (Rylands et al., 2016): the *midas* group: *Saguinus midas* (Linnaeus, 1758), *Saguinus niger* (É. Geoffroy Saint-Hilaire, 1803), *Saguinus ursulus* Hoffmannsegg, 1807; the *bicolor* group: *Saguinus bicolor* (Spix, 1823), *Saguinus martinsi martinsi* (Thomas, 1912), *Saguinus martinsi ochraceus* Hershkovitz, 1966; the *oedipus* group: *Saguinus oedipus* (Linnaeus, 1758), *Saguinus geoffroyi* (Pucheran, 1845), and *Saguinus leucopus* (Günther, 1877); and the *mystax* group: *Saguinus mystax mystax* (Spix, 1823), *Saguinus mystax pileatus* (I. Geoffroy Saint-Hilaire & Deville, 1848), *Saguinus mystax pluto* (Lönnberg, 1926), *Saguinus labiatus labiatus* (É. Geoffroy Saint-Hilaire, 1812), *Saguinus labiatus thomasi* (Goeldi, 1907), *Saguinus labiatus rufiventer* (Gray, 1843), *Saguinus imperator imperator* (Goeldi, 1907), *Saguinus imperator subgrisescens* (Lönnberg, 1940), and *Saguinus inustus* (Schwarz, 1951).

**Figure 2:**
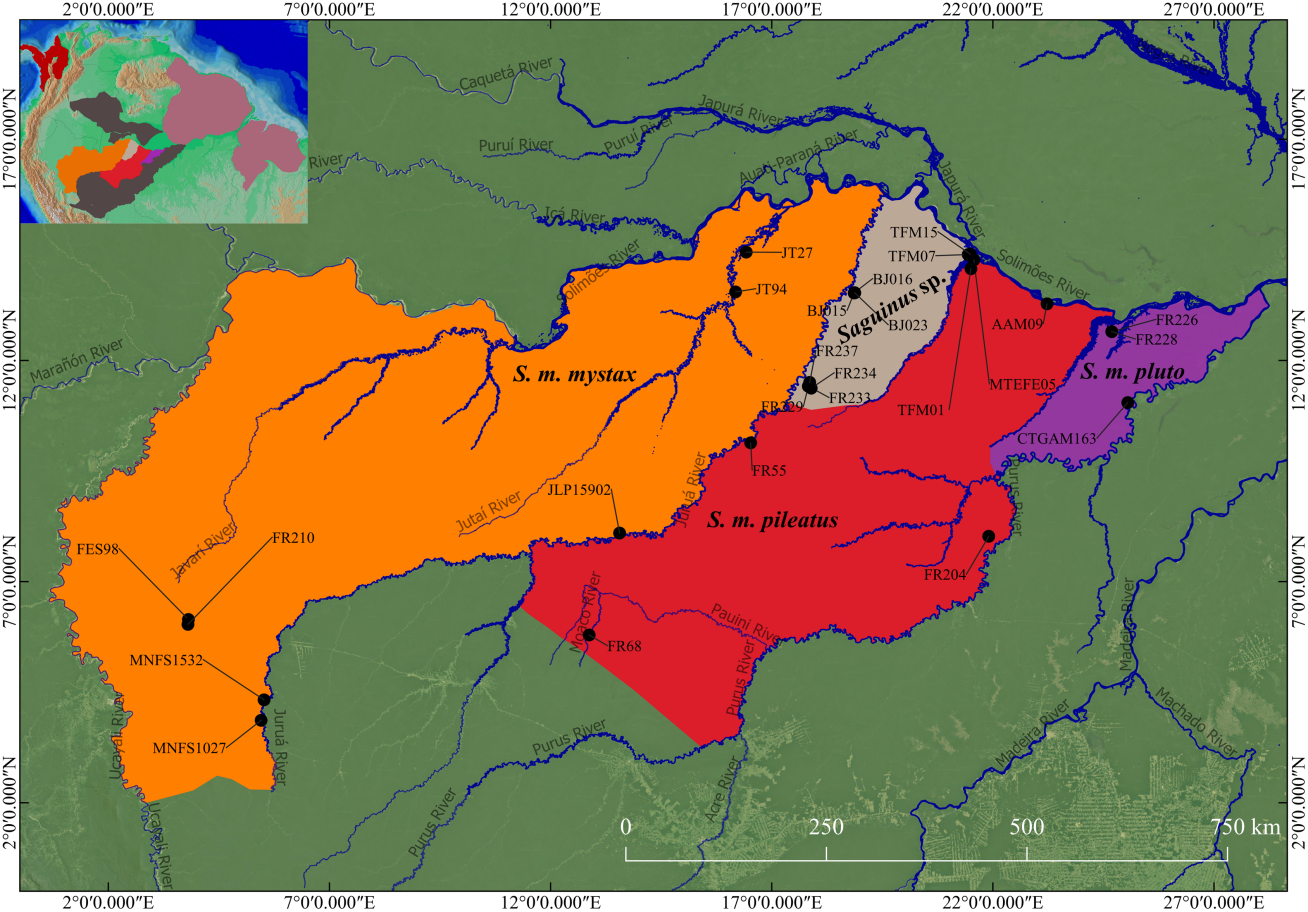
Geographic distribution of *S*. *mystax* subspecies and *Saguinus* sp. Black circles indicate location of genetic samples. Polygons modified from IUCN and Sampaio, Rohe & Rylands (2018). Blue lines represent Amazonian rivers. The small map on superior side left shows the North of South America, and the distribution of *Saguinus* three species groups.

The *bicolor*, *midas* and *oedipus* groups have been subject to taxonomic revisions (Hanihara & Natori, 1987; Kanazawa & Rosenberger, 1988; Skinner, 1991; Natori & Hanihara, 1992; Moore & Cheverud, 1992; Rylands, 1993; Vallinoto et al., 2006; Rylands & Mittermeier, 2009; Gregorin & Vivo, 2013) since the seminal study of Hershkovitz (1977). In contrast, the taxonomy of the *mystax* group has remained practically unchanged, and current taxonomy follows Hershkovitz (1977) who considered these taxa geographic races of the same species, hence relegating them to the subspecies status (Rylands et al., 2016). However, Groves (2001) noted that while *S*. *m*. *mystax* and *S*. *m*. *pluto* are quite similar to each other, *S*. *m*. *pileatus* is phenotypically divergent and therefore opinionated that it likely is a species. Following this logic, Rylands & Mittermeier (2009) opinionated that *S. m. mystax* and *S. m. pluto* are likely different species given that their areas of occurrence are separated by that of *S. m. pileatus* (Fig. 2). Taxonomic clarity and resolution of this issue has, however, not advanced due to lack of specimens available for taxonomic analyses.

**Figure 3:**
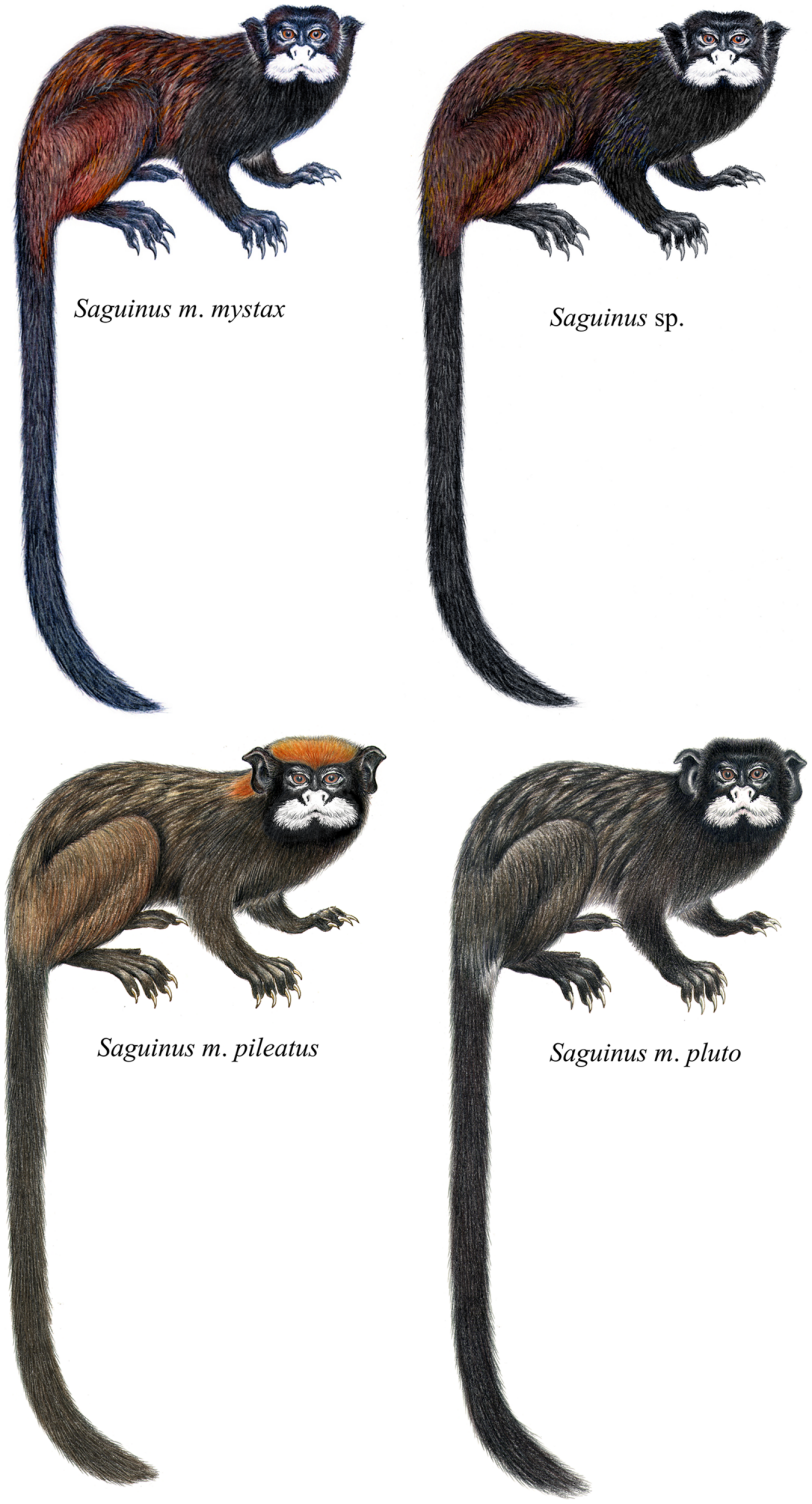
Coat color pattern of *Saguinus mystax* and *Saguinus sp*. analyzed in this study. Illustrations by Stephen D. Nash.

**Figure 4:**
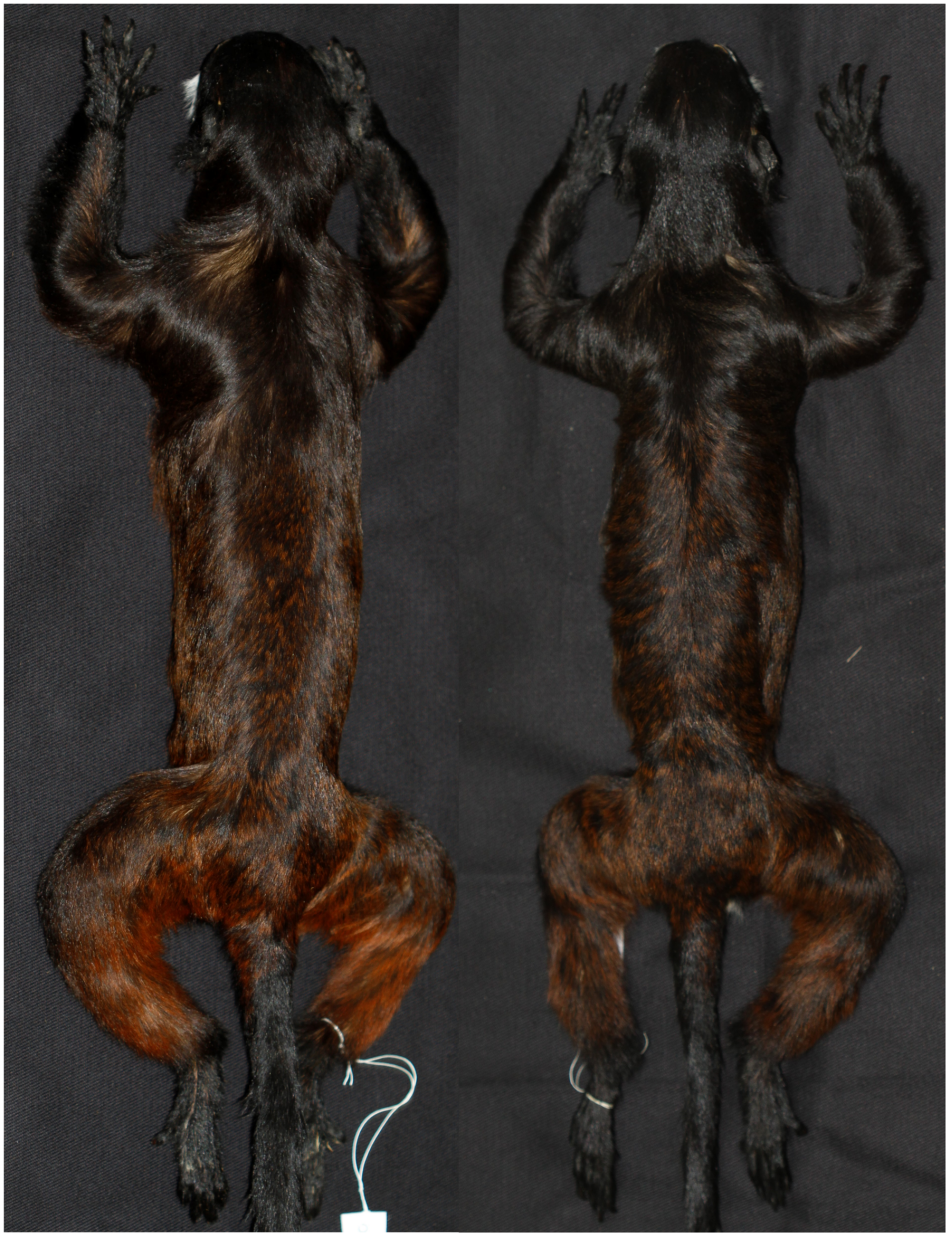
Dorsal views of skins of *Saguinus mystax* (left; IDSM03594) and *Saguinus* sp. (right; IDSM00067).

To clarify the taxonomy of subspecies of *Saguinus mystax*, we carried out extensive field surveys in their areas of distribution. During field surveys in the Juruá-Tefé interfluve, we observed that specimens of *S*. *m*. *mystax* from the right bank of the Juruá River differed in phenotype from the specimens from the left bank of this river (Figs. 3 and 4).

Considering the above, in this study we test the taxonomic hypothesis that the described taxa of *S*. *mystax* and the new morphotype are independent evolutionary lineages, *i.e*., species (the General Lineage Species Concept of de Queiroz, 1999, 2007). To this end, we generated double digest restriction associated DNA (ddRAD) data and used these data to conduct coalescent-based tests of taxonomic hypotheses (Fujita et al., 2012; Leaché et al., 2014). Subsequently we evaluated the results of these analyses in light of phenotypic coat color data and geographic distributions of the analyzed taxa.

## Materials and Methods

### Specimen sampling

All analyzed specimens originated from museum collections. This study did not motivate the collection of these specimens. All specimens were collected under licenses granted by the Instituto Chico Mendes de Conservação da Biodiversidade/Sistema de Autorização e Informação em Biodiversidade and in accordance with the ethical recommendations of the Conselho Federal de Biologia (CFBio; Federal Council of Biologists), Resolution 301 (December 8, 2012).

### Collection of phenotypic data

Using Hershkovitz’s (1968, 1977) framework of chromogenetic fields (Fig. S1), we collected phenotypic data from specimens of *S*. *m*. *mystax*, *S*. *m*. *pluto*, *S*. *m*. *pileatus*, and *Saguinus* sp. Chromogenetic fields are defined as consistently distinct areas of the body identifiable by hair color and color patterns of the coat (Hershkovitz, 1977). Chromogenic fields have been found to be informative in taxonomic studies of callitrichids (Hershkovitz, 1968, 1977; Vivo, 1991; Mittermeier, Schwarz & Ayres, 1992; Silva & Noronha, 1998; Rohe et al., 2009; Sampaio et al., 2015; Garbino, 2015; Garbino & Martins-Junior, 2018; Costa-Araújo et al., 2019, 2021).

For each specimen we collected color and color pattern characteristics of each of the 11 chromogenetic fields. We considered color tones as character states. All data were collected in daylight conditions using the color codes of the Munsell Color (2000) and are summarized by taxon in Table 1. We examined 38 specimens deposited in the collections of the Instituto Nacional de Pesquisas da Amazônia (INPA) and the Instituto de Desenvolvimento Sustentável Mamirauá (IDSM); of these, 25 specimens were included in molecular analyses (Table S1).

**Table 1:** Coat color pattern of taxa of the *Saguinus mystax* complex based on our specimen’s examination.

Chromogenetic fields	S. *m*. *mystax*	*Saguinus* sp.	S. *m*. *pileatus*	S. *m*. *pluto*
I. Forehead	Light black	Light black	Reddish orange	Black
II. Crown	Light black	**Light black brown**	Reddish orange	Black
III. Temporal patch	Black	Black	Black	Black
IV. Mantle	Light black brown, subterminal band is orange, terminal band is black	**Light black brown, subterminal band is yellow, terminal band is black**	Light black brown with fine light brownish yellow or orange ticking	Black and ticked with light brownish yellow
V. Throat	Black	Black	Light black or brown	Black
VI. Chest	Black	Light black	Light black or brown	Light black
VII. Forelimbs	Black	**Light black brown, subterminal band is yellow, terminal band is black**	Light black	Light black and light brownish yellow
VIII. Saddle	Light black with orange	**Light black with brown**	Light black brown and light brownish yellow	Light black and light brownish yellow
IX. Rump	Light black with orange	**Light black with brown**	Light black brown and light brownish yellow	Light black and light brownish yellow
X. Hindlimbs	Light black with orange	**Light black with brown**	Light black	Light black and light brownish yellow
XI. Tail	Black	Black	Black	Black

**Note:**

Characters unique to *Saguinus* sp. are marked in bold.

### Collection of ddRAD data

We extracted DNA from tissues deposited at INPA, IDSM, and the Universidade Federal do Amazonas (UFAM). We extracted whole genomic DNA from tissue samples using the standard phenol-chloroform extraction protocol (Sambrook & Russell, 2001). We then generated ddRAD sequence data (Peterson et al., 2012) using a modified protocol optimized for the IonTorrent PGM described in Boubli et al. (2018) (see: https://github.com/legalLab/protocols-scripts). We generated data for 25 specimens of the *Saguinus mystax* species, including representatives of all described subspecies and spanning the known geographic distribution of the taxa within Brazil. We also generated data for three specimens of *Saguinus inustus*, an outgroup taxon. We used the *SdaI* and *Csp6I* restriction enzymes (Thermo Fisher, Waltham, MA, USA), and size selected fragments in the range of 320–400 bp using PippinPrep (Sage Science, Beverly, MA, USA). Samples were sequenced on the IonTorrent PGM using the manufacturer’s recommended protocol.

Sequencing reads were processed using the pyRAD pipeline (Eaton, 2014). During *de novo* assembly, we used a minimum coverage of 6x per locus, assembling all fragments in the 300–400 bp range. Nucleotides with PHRED scores <30 were excluded, and loci with more than six low quality nucleotides were excluded. Subsequently we clustered alleles within loci and loci across individuals, both at a minimum of 88% similarity. Finally, we generated the final dataset by filtering the clusters such that all loci with more than 50% heterozygotes were excluded-likely paralogs, and the locus had to have been present in at least 19 of the 28 specimens. The resulting dataset contained 556 loci and 196,771 nucleotides. We subsequently statistically reduced the 556 loci into 163 partitions using PartitionFinder2 (Lanfear et al. 2017).

We also processed our raw reads using DiscoSnp-RAD (Gauthier et al., 2020), which uses De Bruijn graphs to circumvent the need for clustering of reads, reducing data loss due to low coverage within individuals. We extracted single nucleotide polymorphisms (SNPs) from our reads using a minimum read depth of five. Furthermore, we filtered these loci on rank (Gauthier et al., 2020), retaining those SNPs with rank >0.9—a statistic incorporating the discriminant power and read coverage of each SNP—and those that were present in at least 90% of the samples, *i.e*., no more than two individuals had missing data at any particular SNP locus. The resulting variant call file (VCF) was filtered to retain all loci with 2–5 SNPs per locus following Malinsky et al. (2018) resulting in a 98.38% complete matrix comprising 158 uninked loci and 351 SNPs. We also filtered the original VCF file to sample the highest quality SNP from each locus, generating a VCF of unlinked loci. The resulting matrix was 98.55% complete and comprised 4,550 unlinked loci.

### Clustering analyses

An initial population structure analysis was performed using fineRADstructure (Malinsky et al., 2018) (Fig. S2). This method takes full advantage of ddRAD sequences by using all linked SNPs present at each locus to generate a co-ancestry table, and then uses Markov chains within a Bayesian framework to retrieve fine-scale groupings of individuals from this table. The MCMC chain for group definition was formed from 1 million steps, sampling every 1000th step, preceded by 1 million of burn-in steps. Convergence of the MCMC chain was verified visually.

### Phylogenetic analyses

We used ASTRAL-III (Zhang et al., 2018) for phylogenetic reconstruction because it is fast, consistent with missing data (Nute et al., 2018) and considers the historical independence of different loci in a framework that takes incomplete lineage sorting into account (ILS). Furthermore, since ASTRAL-III constructs a supertree from pre-estimated gene trees, these can be inferred using the complete information contained in the ddRAD sequences. The estimation of gene trees was performed with RAxML (Stamatakis, 2014) using the Gamma GTR model of molecular evolution, from the best of 10 independent estimates of maximum likelihood trees and support values estimated from 100 bootstraps. Finally, before estimating the supertree in ASTRAL-III, following Zhang, Sayyari & Mirarab (2017) and Mirarab (2019) nodes with bootstrap support less than 10 were collapsed into polytomies using Newick Utilities (Junier & Zdobnov, 2010).

We also estimated a phylogeny using SVDquartets (Chifman & Kubatko, 2014) implemented in PAUP (Swofford, 2002). This method infers quartets based on summaries of SNPs in a concatenated sequence matrix of species using a full coalescent model. Sequences of *Saguinus inustus* were used as outgroup. We opted to use both methods since although both are fully coalescent methods of phylogenetic reconstruction, they have slightly different premises and use data in a different form (sequences *vs* SNPs).

### Tests of taxonomic hypotheses

We carried out a path sampling analysis in BEAST2 (Bouckaert et al., 2014) testing competing taxonomic hypotheses derived from current taxonomy, our phylogenomic analyses and pelage color patterns. The potential taxa whose taxonomic status was tested were *S. m. mystax*, *S. m. pileatus*, *S. m. pluto* and *Saguinus* sp. The taxonomic hypotheses were: (1) one species—(*S*. *m*. *mystax* + *S*. *m*. *pileatus* + *S*. *m*. *pluto* + *Saguinus* sp.); (2) two species—(*S*. *m*. *mystax* + *Saguinus* sp., *S*. *m*. *pileatus* + *S*. *m*. *pluto*); (3) three species—(*S*. *m*. *mystax* + *Saguinus* sp., *S*. *m*. *pileatus*, *S*. *m*. *pluto*); (4) three species—(*S*. *m*. *mystax*, *Saguinus* sp., *S*. *m*. *pileatus* + *S*. *m*. *pluto*); (5) four species—(*S*. *m*. *mystax*, *Saguinus* sp., *S*. *m*. *pileatus*, *S*. *m*. *pluto*). Marginal probabilities of the competing taxonomic hypotheses were then compared by Bayes factors (Kass & Raftery, 1995).

## Results

### Phenotypic analysis

*Saguinus m*. *mystax*, *S*. *m*. *pileatus*, *S*. *m*. *pluto* and *Saguinus* sp. were diagnosable by pelage color characters (Table 1; Fig. 3). Specimens of *Saguinus* sp. from the Juruá-Tefé interfluve showed a distinct set of diagnostic characters that differentiated these specimens from *Saguinus m*. *mystax* (Table 1; Figs. 3 and 4).

### Phylogenetic analyses

**Figure 5:**
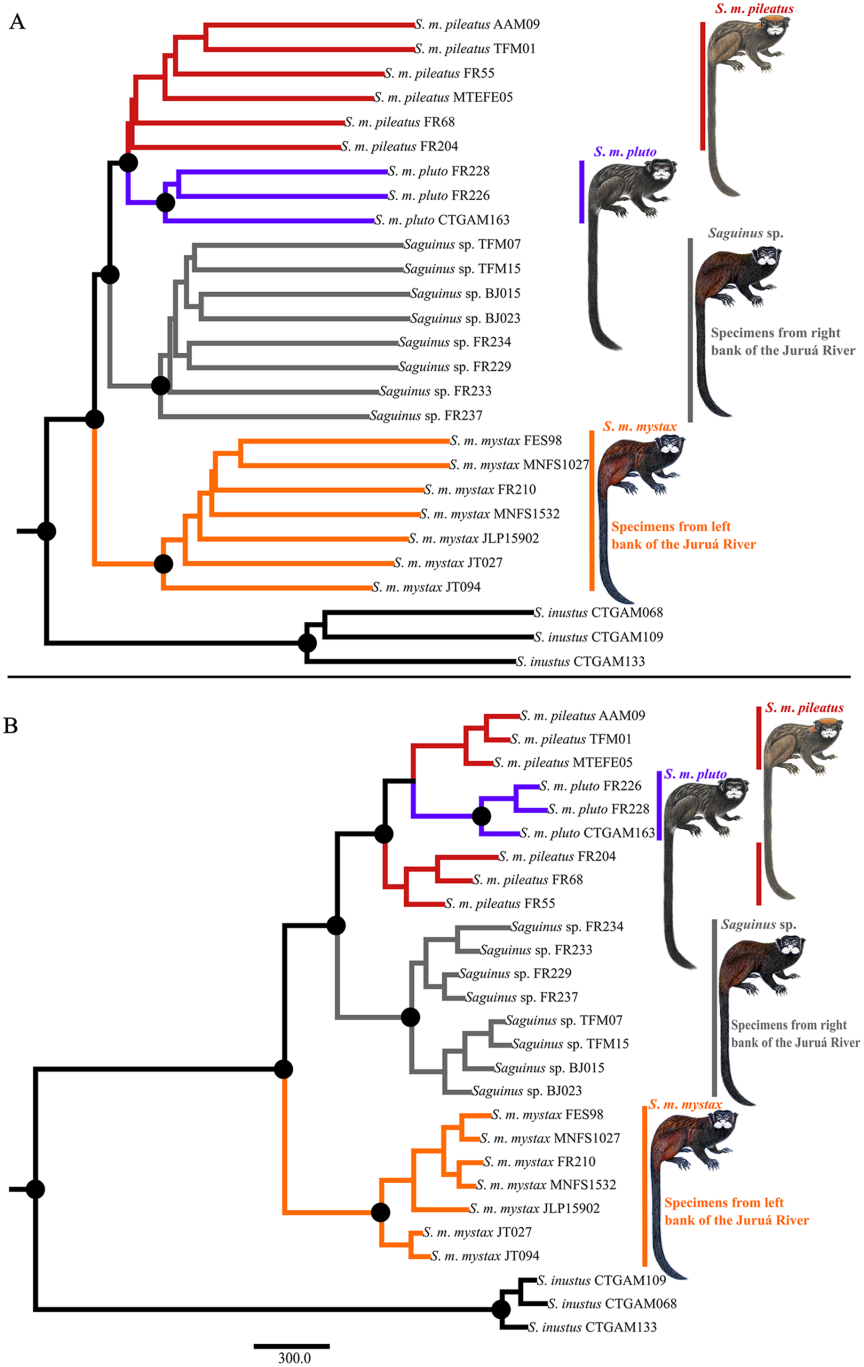
Phylogeny of *Saguinus mystax*. (A) ASTRAL-III phylogeny. (B) SVDquartet phylogeny. Black dots on major clades indicate supported clades *sensu* Hillis & Bull (1993)—support values >75%.

Phylogenetic analyses carried out in ASTRAL-III and SVDQuartets were concordant and recovered three well-supported clades representing *S*. *m*. *mystax* from the left bank of the Juruá River, a clade from the right bank of the Juruá River—the Juruá-Tefé interfluve representing *Saguinus* sp., and a clade comprising *S*. *m*. *pileatus* and *S*. *m*. *pluto* (Fig. 5A). In the SVDQuartets analysis *S*. *m*. *pluto* was nested within *S*. *m*. *pileatus* (Fig. 5B), while in the ASTRAL-III analysis *S*. *m*. *pileatus* and *S*. *m*. *pluto* were recovered as monophyletic, however, the monophyly of *S. m. pileatus* was not supported (Fig. 5A).

### Tests of taxonomic hypotheses

Taxonomic hypothesis testing indicated highest support for the existence of three species: *S*. *m*. *mystax*, *Saguinus* sp., and *S*. *m*. *pileatus* + *S*. *m*. *pluto*. Considering that a Bayes factor >10 is decisive (Kass & Raftery, 1995), the four species hypothesis (*S*. *m*. *mystax*, *Saguinus* sp., *S*. *m*. *pileatus*, *S*. *m*. *pluto*) was strongly rejected (BF = 100.92), as was the three species hypothesis compatible with current subspecific classification (*S*. *m*. *mystax* + *Saguinus* sp., *S*. *m*. *pileatus*, *S*. *m*. *pluto*) (BF = 116.64). All other taxonomic hypotheses were strongly rejected as well (Table 2).

**Table 2:** Test of taxonomic hypotheses using Path Sampling in BEAST2.

Hypothesis	Marginal likelihood	Bayes factor
One species* (*mystax*+sp.+*pileatus*+*pluto*)	−313,201.0512676738	278.979
Two species^¤^ (*mystax*, sp.+*pileatus*+*pluto*)	−313,099.0317716367	65.801
Three species^§^ (*mystax*, sp., *pileatus*+*pluto*)	−313,061.5617465277	–
Three species^‡^ (*mystax*+sp., *pileatus*, *pluto*)	−313,119.88354474324	116.6436
Four species^†^ (*mystax*, sp., *pileatus*, *pluto*)	−313,112.0196339431	100.9158

**Notes:**

Differences in marginal likelihoods were evaluated using Bayes Factors. The three species (mystax, sp, pileatus+pluto) taxonomic hypothesis has the highest marginal likelihood and all other taxonomic hypotheses are decisively less likely.

* Current species hypothesis—*Saguinus mystax*.

¤ Conservative taxonomic hypothesis suggested two main clades of *Saguinus mystax*.

§ Taxonomic hypothesis suggested by ASTRAL and SVDquartet analyses.

‡ Current taxonomic hypothesis—one species with three subspecies.

† Taxonomic hypothesis suggested by coat color and color pattern characters—four taxa.

The hypothesis that the subspecies of *Saguinus mystax* may represent taxa from independent evolutionary lineages is supported by phenotypic diagnoses and allopatric occurrence of the species. While *S*. *m*. *pileatus* and *S*. *m*. *pluto* were not supported as species, both taxa are morphologically diagnosable and are allopatrically distributed. Therefore, conservatively and in light of taxonomic stability, we maintain these two taxa as subspecies, until we can analyze additional geographically representative samples of *S*. *m*. *pluto*. The taxon *S*. *m*. *pileatus* was described by Saint-Hilaire & Deville in 1848 while the taxon *S*. *m*. *pluto* was described by Lönnberg in 1926. Consequently, the epithet *pileatus* takes precedence over *pluto* (International Commission on Zoological Nomenclature, 1999).

**Taxonomy**

Order Primates Linnaeus, 1758

Family Callitrichidae Thomas, 1903

Genus *Saguinus* Hoffmannsegg, 1807

***Saguinus mystax* (Spix, 1823)**

**Common name:** Spix’s Mustached Tamarin

**Description.** Head light-black, skin of circumnarial and circumbucal area except at symphysis unpigmented and covered with comparatively long white hairs, whiskers well-developed, remainder of face pigmented or unpigmented and covered with black hairs. Mantle blackish brown with orange subterminal band, terminal band is black, basal one-fourth to one-half of hairs white and showing through irregularly at surface. Arms black, hairs of saddle, rump, and outer side of thighs light black with orange or ochraceus orange subterminal band. Lateral fringe like saddle, but basal portion of hairs drab or withish, and upper surface of hands and feet black. Underparts from middle of lower lip to belly and inner sides of limbs black to blackish brown. The tail is black, except the base that is like the rump. External genitalia mostly or entirely unpigmented and sparsely covered white hairs. We observed tonal variation in saddle, rump and hindlimbs in some individuals of *S*. *mystax*. This variation was attributed to individual variation.

**Type locality:** Near São Paulo de Olivença, south bank of the Solimões/Amazonas River, Amazonas, Brazil.

**Synonyms**

*Midas mystax* Spix, J.B. von. 1823. Sim. Vespert. Brasil., p. 29. pl. 22.

*Jacchus labiatus* Poeppig, E.F. 1831. Froriep Not. 32: 148.

*H*[*apale*] *mystax* Wagner, J.A. 1855. Schreber’s Säugth. Suppl. 5: 129.

*Jacchus mystax* Gerrard, 1862. Cat. Bones Brit. Mus., p. 29.

*M*[*idas*] *labiatus* Reichenbach, H.G.L. 1862. Vollst. Naturg. Affen, p.11, pl. 3.

*L*[*eontocebus*] *mystax* Cabrera, A. 1912. Trab. Mus. Cienc. Nat., Madrid (11): 29.

*Hapale* (*Leontocebus*) *mystax* Lampert, H. 1926. Morph Jahrb., 55: 611.

[*Mystax*] *mystax* Pocock, R.I. 1917. Ann. Mag. Nat. Hist., ser. 8, 20: 117.

*Mystax mystax* Thomas, O. 1928. Ann. Mag. Nat. Hist., ser. 10, 2: 255.

*Tamarin mystax* Cruz Lima, E. 1945. Mamíferos da Amazônia. 1. Primates, p. 220, pl. 37.

*Marikina mystax* Hershkovitz, P. 1949. Proc. U. S. Nat. Mus. 98: 412.

*Tamarinus mystax* Hill, W.C.O. 1957. Primates. Comp. Anat. Taxon. III. Pithecoidea Platyrrhini (Fam. Hapalidae and Callimiconidae). Edinburgh Univ. Press, Edinburgh, p. 164.

*L*[*eontocebus*] *mystax*. Hill, W.C.O. 1961. Proc. Zool. Soc. Lond. 137(2): 321.

*Saguinus mystax* Anderson, E.T., Lewis, J.P., Passovoy, M. & Trobaugh, F.E. 1967. Lab. Anim. Care. 17(1): 37.

*Saguinus mystax mystax* Hershkovitz, P. 1968. Evolution 22(3): 563.

*Saguinus* (*Tamarinus*) *mystax mystax* Garbino, G.S.T. & Martins-Junior, A.M.G. 2018. Mol. Phylogenet. Evol. 118: 169.

**Distribution.** *Saguinus mystax* occurs in Peru and Brazil (Fig. 2).

The distribution of this species is south of the Solimões/Amazonas River, from the left bank of the Juruá River (Hershkovitz, 1977; Johns, 1985; Rylands, Coimbra-Filho & Mittermeier, 1993; Heymann et al., 2021), the western boundary is the Ucayali River. To the south the limit is the Blanco River in Peru (Hershkovitz, 1977; Heymann et al., 2021).

**Specimens Examined** (*N* = 13): Brazil: JT021 (IDSM00039), JT025 (IDSM00004), JT027 (IDSM00045), JT052 (IDSM00067), JT055 (IDSM00067), JT084 (IDSM00776), JT094 (IDSM00778), MNFS1027 (INPA), MNFS1532 (INPA), FES98 (IDSM03681), FR210 (INPA), JLP15902 (INPA).

***Saguinus pileatus*** ***pileatus* (I. Geoffroy Saint-Hilaire & Deville, 1848) comb. nov.**

**Common name:** Red-capped Mustached Tamarin

**Description.** Forehead and crown with broad posteriorly bifurcated reddish orange cap, the color extending forward as a thin line between orbits. Superciliary region, cheeks, temples, and interorbital space black. Hairs of midline of muzzle forming a low crest, black anteriorly, light red posteriorly, the color continuous with that of mid-frontal region. Skin of circumnarial and circumbucal areas except at symphysis unpigmented and covered with comparatively long white hairs forming whiskers, remainder of facial skin more or less pigmented and covered with black hair. Mantle blackish brown with fine light brownish yellow or orange, the basal one-fourth to one-half of the hairs drab to brownish black along midline. Saddle and rump light brownish yellow subterminal band. Dark brown hairs of lateral fringe faintly or not at all banded subterminally, pale brown, drab, or white basally. Outer sides of hindlimbs brown, upper surface of hands and feet is light black. Ventral surface from middle of lower lip to be belly and inner sides of limbs, blackish brown. The tail is black, except the base that is like the rump.

**Type locality:** Lago de Tefé, near its mouth at Solimões/Amazonas River, Amazonas Brazil (Hershkovitz, 1977, p. 699).

**Synonyms**

*Midas pileatus* Geoffroy Saint-Hilaire, I. & Deville, É., 1848. Compt. Rend. Acad. Sci., Paris 27: 499.

*Hapale pileata* Gervais, F. L. P. 1854. Hist. Nat. Mamm. 1: 152.

*Midas pileatus juruanus* Ihering, H. von. 1904. Rev. Mus. Paulista, São Paulo 6: 416. Type locality: João Pessoa, upper Juruá River, Amazonas, Brazil.

*Leontocebus pileatus* Elliot, D.G. 1913. A Review of the Primates 1: 197.

*Tamarin pileatus* Cruz Lima, E. 1945. Mamíferos da Amazônia. 1. Primates, p. 228.

*Marikina pileata juruana* Hershkovitz, P. 1949. Proc. U. S. Nat. Mus. 98: 413.

*Marikina pileata pileata* Hershkovitz, P. 1949. Proc. U. S. Nat. Mus. 98: 413.

*Tamarinus pileatus* Hill, W.C.O. 1957. Primates. Comp. Anat. Taxon. III. Pithecoidea Platyrrhini (Fam. Hapalidae and Callimiconidae). Edinburgh Univ. Press, Edinburgh, p. 218.

*T*[*amarinus*] *p*[*ileatus*] *juruanus* Hill, W.C.O. 1957. Primates. Comp. Anat. Taxon. III. Pithecoidea Platyrrhini (Fam. Hapalidae and Callimiconidae). Edinburgh Univ. Press, Edinburgh, p. 235.

*Saguinus mystax pileatus* Hill, W.C.O 1957. Primates. Comp. Anat. Taxon. III. Pithecoidea Platyrrhini (Fam. Hapalidae and Callimiconidae). Edinburgh Univ. Press, Edinburgh, p. 235.

*Saguinus mystax pileatus* Hershkovitz, P. 1968. Evolution 22(3): 563.

*Saguinus pileatus* Groves, C.P. 2001. Primate Taxonomy. Smithson. Inst. Press, Washington, DC, p.143.

*Saguinus* (*Tamarinus*) *mystax pileatus*. Garbino, G.S.T. & Martins-Junior, A.M.G. 2018. *Mol. Phylogenet. Evol*. 118: 169.

**Distribution.** *Saguinus pileatus pileatus* is endemic to Brazil. Its distribution occurs south of the Solimões/Amazonas River. The northern portion of its distribution is limited by the Tefé River to the west and the Coari River to the east (Hershkovitz, 1977; Johns, 1985; Rylands, Coimbra-Filho & Mittermeier, 1993; Ravetta & Rohe, 2020a, Fig. 2). The rest of the distribution, above the headwaters of the Coari River, extends to the left bank of the Purus River. Above the headwaters of the Tefé River, the western limit of the distribution is the right bank of the Juruá River (Rylands, Coimbra-Filho & Mittermeier, 1993). *Saguinus pileatus pileatus* also occurs on the left bank of the Tefé River, in the region of the headwaters of this river (Hershkovitz, 1977). To the south, its distribution extends beyond the right bank of the Pauini River (Sampaio, Rohe & Rylands, 2018). In this portion, its distribution seems to be limited by the occurrence of *S*. *i*. *subgrisescens*.

**Specimens Examined** (*N* = 11): Brazil: AAM9 (INPA), FR55 (INPA5684), FR68 (INPA5687), FR204 (INPA), MTEFE02 (IDSM03511), MTEFE05 (IDSM03680), TFM01 (IDSM03384), TFM03 (IDSM02229), TFM06 (IDSM03384), TFM09 (IDSM03654), TFM13 (IDSM03657).

***Saguinus pileatus*** ***pluto* (Lönnberg, 1926) comb. nov.**

**Common name:** White-rumped Mustached Tamarin

**Description.** Head black, skin of circumnarial and circumbucal area except at symphysis unpigmented and covered with hair forming proeminent whiskers, remainder of face covered with black hairs. Mantle black with light brownish yellow, basal portions of hairs drab. Arms black, rump and outer sides of thighs like mantle but with a more even mixture or vermiculation of light black and light brownish yellow. Lateral fringe like back, but hairs drab to grayish basally. Upper surface of the hands and feet black. Underparts from midline of lower lips to anterior part of belly and inner sides of limbs black to blackish brown, lower belly, hairs of inguinal, genital and circumanal region and contiguous portions of the ventral base of the tail is white. Tail is black, except the dorsal portion of the base that is like the rump.

**Type locality:** Brazil: Lago Ayapuá, Purus River, Amazonas (Hershkovitz, 1977; Rylands, Coimbra-Filho & Mittermeier, 1993).

**Synonyms**

*Mystax pluto* Lönnberg, E. 1926. Ark. Zool., Stockholm 18 B(9): 1.

*Tamarin pluto* Elliot, D.G. 1913. A Review of the Primates 1: 230.

*Marikina pluto* Hershkovitz, P. 1949. Proc. U. S. Nat. Mus. 98: 413.

*Marikina* (*Tamarin*) *pluto* Vieira, C.O.C. 1955. Arq. Zool. São Paulo 8: 396.

*Tamarinus pluto* Hill, W.C.O. 1957. Primates. Comp. Anat. Taxon. III. Pithecoidea Platyrrhini (Fam. Hapalidae and Callimiconidae). Edinburgh Univ. Press, Edinburgh, p. 218.

*Leontocebus* (*Leontocebus*) *pluto* Cabrera, A. 1958. Rev. Mus. Argentino Cienc. Nat. “Bernardino Rivadavia” 4(1): 197.

*Saguinus mystax pluto* Hershkovitz, P. 1968. Evolution 22(3): 563. Also, Groves, C. P. 2001. Primate Taxonomy. Smithson. Inst. Press, Washington, DC, p.143.

*Saguinus* (*Tamarinus*) *mystax pluto* Garbino, G.S.T. & Martins-Junior, A.M.G. 2018. Mol. Phylogenet. Evol. 118: 169.

**Distribution.** *Saguinus pileatus pluto* is endemic to Brazil. It occurs south of the Solimões/Amazonas River, from the right bank of the Coari River to the left bank of the Purus River. The southern limit is the Tapauá River (Hershkovitz, 1977; Rylands, Coimbra-Filho & Mittermeier, 1993; Ravetta & Rohe, 2020b, Fig. 2).

**Specimens examined** (*N* = 3): Brazil: CTGA-M163 (UFAM), FR226 (INPA), FR228 (INPA).

***Saguinus kulina* sp. n.** Lopes, Rohe, Bertuol, Polo, Lima, Valsecchi, Santos, Nash, Silva, Boubli, Farias & Hrbek, 2022.

urn:lsid:zoobank.org:act:41E33066-C337-4EA2-AF60-AF75F032E5C.

**Common name:** Kulinas’ tamarin (English), sauim-dos-kulinas (Portuguese), pishi (Kulina).

**Holotype.** IDSM03594, field number BJ015, adult male, skin, skull, skeleton. Specimen collected on July 18th, 2018, in terra firme forest, on the right bank of Andirá River, right bank affluent of the Juruá River, Juruá Municipality, Amazonas State, Brazil.

**Paratopotype.** IDSM03595, field number BJ016, adult female, skin, skull, skeleton. Specimen collected on July 18th, 2018, in terra firme forest, on the right bank of Andirá River, right bank affluent of the Juruá River, Juruá Municipality, Amazonas State, Brazil.

**Paratypes.** IDSM03602, field number BJ023, adult female, skin, skull, skeleton. Specimen collected on July 27th, 2018, in terra firme forest, at Cumaru community, on the left bank of Andirá River, right bank affluent of the Juruá River, Juruá Municipality, Amazonas State, Brazil. IDSM03652, field number TFM07, adult female, skin, skull, skeleton. Specimen collected on August 15th, 2018, in terra firme forest, at Nogueira community, on the left bank of Tefé River, Alvarães Municipality, Amazonas State, Brazil. IDSM03659, field number TFM15, adult female, skin, skull, skeleton. Specimen collected on July 30th, 2019, in terra firme forest, at Nogueira community, on the left bank of Tefé River, Alvarães Municipality, Amazonas State, Brazil.

**Type locality.** Reserva Extrativista do Baixo Juruá (−3.82633S; −66.07572), right bank of Andirá River, Amazonas, Brazil.

**Diagnostic characters (Fig. 3).** The new species is diagnosable from all other species of *Saguinus* by mantle and forelimbs light black brown subterminal band yellow, saddle, rump and hindlimbs light black with brown (Table 1; Figs. 3 and 4).

**Description.** Forehead light black and crown blackish brown, skin of circumbucal and circumnarial area except at symphysis unpigmented and covered with comparatively long white hairs, whiskers well-developed, remainder of face pigmented or unpigmented and covered with black hairs. Mantle light black with yellow subterminal band, terminal band black, the basal one-fourth to one-half of the hairs white and showing through irregularly at surface, the forelimbs are like the mantle up to the elbow. Saddle, rump, and outer side of thighs light black with brown subterminal band, terminal band black. Lateral fringe same as saddle, but basal portion of hairs whitish, and upper surface of hands and feet black. Underparts from middle of lower lip to belly and inner sides of limbs black to light black brown. The tail black, except base which is the same as rump. External genitalia mostly or entirely unpigmented and sparsely covered white hairs.

**Comparisons with closely related species.** In *Saguinus kulina* sp. n. mantle light black with subterminal hairs band yellow, terminal band is black, saddle, rump, hindlimbs are light black with brow, lateral fringe like saddle. In *S*. *mystax* mantle is light black with orange or ochraceus orange subterminal band, and terminal band is black, forelimbs are black, saddle, rump and hindlimbs are light black with orange. In *S*. *p*. *pileatus* forehead and crown with broad posteriorly bifurcated reddish orange cap, the color extending forward as a thin line between orbits, hairs of midline of muzzle forming a low crest, black anteriorly, light red posteriorly, the color continuous with that of mid-frontal region, mantle blackish brown with fine light brownish yellow or orange ticking. In *S*. *p*. *pluto* mantle is black and light brownish yellow, basal portions of hairs drab, rump and outer sides of thighs like mantle but with a more even mixture or vermiculation of light black and light brownish yellow, and circumanal region whitish.

**Etymology.** The specific name is a noun in apposition and honors the Kulina indigenous peoples of the Kumaru Indigenous Territory, the largest indigenous territory within Juruá–Tefé interfluve.

**Geographic Distribution.** *Saguinus kulina* sp. n. is endemic to the western Amazonia, occurring between the right bank of the Juruá River and the left bank of the Tefé River (Fig. 2).

**Synonyms**

*Midas mystax* Spix, J.B. von. 1823. Sim. Vespert. Brasil., p. 29. pl. 22.

*Jacchus labiatus* Poeppig, E.F. 1831. Froriep Not. 32: 148.

*H*[*apale*] *mystax* Wagner, J.A. 1855. Schreber’s Säugth. Suppl. 5: 129.

*Jacchus mystax* Gerrard, 1862. Cat. Bones Brit. Mus., p. 29.

*M*[*idas*] *labiatus* Reichenbach, H.G.L. 1862. Vollst. Naturg. Affen, p.11, pl. 3.

*L*[*eontocebus*] *mystax* Cabrera, A. 1912. Trab. Mus. Cienc. Nat., Madrid (11): 29.

*Hapale* (*Leontocebus*) *mystax* Lampert, H. 1926. *Morph Jahrb*., 55: 611.

[*Mystax*] *mystax* Pocock, R. I. 1917. Ann. Mag. Nat. Hist., ser. 8, 20: 117.

*Mystax mystax* Thomas, O. 1928. Ann. Mag. Nat. Hist., ser. 10, 2: 255.

*Tamarin mystax* Cruz Lima, E. 1945. Mamíferos da Amazônia. 1. Primates, p. 220, pl. 37.

*Marikina mystax* Hershkovitz, P. 1949. Proc. U. S. Nat. Mus. 98: 412.

*Tamarinus mystax* Hill, W.C.O. 1957. Primates. Comp. Anat. Taxon. III. Pithecoidea Platyrrhini (Fam. Hapalidae and Callimiconidae). Edinburgh Univ. Press, Edinburgh, p. 164.

*L*[*eontocebus*] *mystax*. Hill, W.C.O. 1961. Proc. Zool. Soc. Lond. 137(2): 321.

*Saguinus mystax* Anderson, E.T., Lewis, J.P., Passovoy, M. & Trobaugh, F.E. 1967. Lab. Anim. Care. 17(1): 37.

*Saguinus mystax mystax* Hershkovitz, P. 1968. Evolution 22(3): 563.

*Saguinus* (*Tamarinus*) *mystax mystax* Garbino, G.S.T. & Martins-Junior, A.M.G. 2018. Mol. Phylogenet. Evol. 118: 169.

**Specimens examined** (*N* = 9): Brazil: BJ015 (IDSM03594), BJ016 (IDSM03595), BJ023 (IDSM03602), FR229 (INPA), FR233 (INPA), FR234 (INPA), FR237 (INPA), TFM07 (IDSM03652), TFM15 (IDSM03659).

The electronic version of this article in portable document format will represent a published work according to the International Commission on Zoological Nomenclature (ICZN), and hence the new name contained in the electronic version is effectively published under that Code from the electronic edition alone. This published work and the nomenclatural acts it contains have been registered in ZooBank, the online registration system for the ICZN. The ZooBank Life Science Identifiers (LSIDs) can be resolved, and the associated information viewed through any standard web browser by appending the LSID to the prefix http://zoobank.org/. The LSID for this publication is: urn:lsid:zoobank.org:pub:64332121-FA59-4102-9487-C586912500FD. The online version of this work is archived and available from the following digital repositories: PeerJ, PubMed Central, and CLOCKSS.

## Discussion

Recent advances in the collection of genomic data and in the analyses of these data have resulted in renewed interest in taxonomically challenging groups. These advances have resulted in the discovery of new species within not just taxonomically neglected groups, but also of large charismatic mammals such as giraffes (Coimbra et al., 2021) and orangutans (Nater et al., 2017). The application of phylogenetic methods in species description has been criticized as taxonomic inflation by some authors (*e.g*., Isaac, Mallet & Mace, 2004; Mallet, Isaac & Mace, 2005)—much of which can be attributed to the apparent arbitrariness with which clades at different hierarchical levels are delimited as species—however, with the introduction of statistically rigorous coalescent-theory-based methods that permit differentiating between intra- and interspecific patterns of evolution (Fujita et al., 2012; Grummer, Bryson & Reeder, 2014; Leaché et al., 2014) this no longer needs to be an issue. These methods lend themselves to rigorous taxonomic hypothesis testing *via* comparisons on the marginal likelihoods of different taxonomic hypotheses. We can ask, for example, what is the likelihood of an observed pattern of evolution—utilizing our knowledge of patterns of intra- and interspecific coalescence embodied in the coalescent theory—given the existence of one taxon *vs* two taxa. The likelihoods of these two alternate hypotheses can then be compared using the Bayes Factor framework, and the most likely taxonomic hypothesis can be chosen in a non-arbitrary statistically rigorous framework.

Using this conceptual framework, we revised the taxonomy of the *Saguinus mystax* species. Our analyses overwhelmingly support the existence of three species: *Saguinus mystax*, *Saguinus pileatus*, and a third species new to science (*Saguinus kulina* sp. n.). This taxonomic hypothesis is congruent with phenotypic, phylogenetic and distributional data. In recognition of phenotypic differences and for the purpose of nomenclatorial stability, we also maintain the two subspecies *S*. *p*. *pileatus* and *S*. *p. pluto*.

These results in part confirm but also contradict the seminal study of Hershkovitz (1977). Hershkovitz (1977) recognized that *Saguinus mystax* was a taxonomic complex with one of the largest distributions of any callitrichid primate, designating subspecies to accommodate this taxonomic diversity, and proposing distributional limits of these taxa. Although Hershkovitz (1977) examined a large series of *S*. *mystax*, he did not examine any specimens from the Juruá–Tefé interfluve; this is evidenced by his proposal of occurrence of *S*. *p*. *pileatus* in this area. This is not surprising given that specimens of the *Saguinus mystax* species were never collected in the Juruá–Tefé interfluve until this study, and therefore could never have been analyzed. Once collected, the inclusion of these specimens resulted in the discovery of a species previously unknown to science.

Our results also differ in important aspects from Hershkovitz’s (1977) taxonomic proposal. We demonstrated that the taxa *S*. *p*. *pileatus* and *S*. *p. pluto* are not subspecies of *S*. *mystax*, but rather a separate species, a result that is partially in agreement with Groves (2001) who postulated that the subspecies *S*. *p. pileatus* should be elevated to a species level since it is phenotypically divergent from *S*. *mystax*. Our analyses rejected species-level divergence of the taxa *S*. *p*. *pileatus* and *S*. *p*. *pluto* in favor of one species-level taxon *Saguinus pileatus*. This taxonomic conclusion is supported by the SVDquartet phylogeny (Fig. 5B), it is not contradicted by the ASTRAL phylogeny (Fig. 5A), however it is not supported by phenotypic characters and distribution. The taxa *S*. *p*. *pileatus* and *S*. *p*. *pluto* are distributed allopatrically and separated by the Coari River, and also differ in pelage color and patterns. These taxa may be at an early stage of speciation, and therefore we recognized this divergence by maintaining these taxa as subspecies of *Saguinus pileatus*. A better understanding of the evolutionary dynamics and the potential incipient speciation within *Saguinus pileatus* will require additional sampling and field observations, particularly at the southern end of the geographic distribution of *S*. *p*. *pluto* where, at the headwaters of the Coari River, *S*. *p*. *pileatus* and *S*. *p*. *pluto* are hypothesized to come into geographic contact.

The *Saguinus mystax* species group is not the only one in need of taxonomic revision. Much of the taxonomy of Amazonian primates is based on the seminal work of Hershkovitz (1977) and has not changed much in the last 45 years. As genomic data becomes more accessible, including data from centuries-old type specimens (Boubli et al., 2021), and as new biodiversity surveys and scientific collections provide new material for rigorous and biologically realistic analyses, our understanding of the taxonomic diversity of Amazonian primates will change (*e.g*., Boubli et al., 2018, 2019; Costa-Araújo et al., 2019, 2021). These taxonomic changes will not be the consequence of taxonomic inflation, but rather will reflect the underlying evolutionary and speciation processes that have generated and are generating the immense Amazonian biodiversity. Perhaps most importantly these taxonomic revisions will lay the foundation not only for effective science driven conservation but also for understanding the very processes that drove the evolution of Amazonian biodiversity.

## Conclusions

We describe a new species of *Saguinus* from the Juruá–Tefé interfluve based on robust genomic evidence, pelage characters and geographic distribution. We also elevate *Saguinus mystax* to the species level, but we find no conclusive evidence for species level differentiation of *Saguinus p*. *pileatus* and *Saguinus p*. *pluto*. Finally, we emphasize that field surveys and scientific collection of specimens are essential for the continued advancement of knowledge of primate diversity specifically, and all Amazon biodiversity in general.

## Supplemental Information

10.7717/peerj.14526/supp-1 Supplemental Information 1 Representation of a tamarin skin and the pelage pigmentation characters examined.

10.7717/peerj.14526/supp-2 Supplemental Information 2 Clustered fineRADstructure co-ancestry matrix of *Saguinus mystax*. Individuals within the same species/subspecies share more coancestry with each other than other species/subspecies, indicated by colors. Sm = *Saguinus mystax*, Sk = *Saguinus kulina*, Sppl = *Saguinus pileatus pluto*, Sppi = *Saguinus pileatus pileatus*.

10.7717/peerj.14526/supp-3 Supplemental Information 3 Specimens, field number/collection numbers, locality, and geographic coordinates.

## Additional Information and Declarations

### Field Study Permissions

The following information was supplied relating to field study approvals (*i.e*., approving body and any reference numbers): Instituto Chico Mendes de Conservação da Biodiversidade (ICMBio).

### DNA Deposition

The following information was supplied regarding the deposition of DNA sequences: The raw sequence reads are available at Genbank: PRJNA552061.

### Data Availability

The following information was supplied regarding data availability: The raw data is available at: Gerson Paulino Lopes. (2022). Taxonomic review of *Saguinus mystax* (Spix, 1823) (Primates, Callitrichidae), and description of a new species (In review) [Data set]. Zenodo. https://doi.org/10.5281/zenodo.7093175.

### New Species Registration

The following information was supplied regarding the registration of a newly described species: Saguinus kulina species LSID: urn:lsid:zoobank.org:act:41E33066-C337-4EA2-AF60-AF75F032E5CD. Publication LSID: urn:lsid:zoobank.org:pub:64332121-FA59-4102-9487-C586912500FD.

## References

[ref-1] Boubli JP, Byrne H, Silva MNF, Silva-Júnior J, Araújo RC, Bertuol F, Gonçalves J, Melo FR, Rylands AB, Mittermeier RA, Silva FE, Nash SD, Canale G, Alencar RM, Rossi RV, Carneiro J, Sampaio I, Farias IP, Schneider H, Hrbek T (2019). On a new species of titi monkey (Primates: *Plecturocebus* Byrne et al., 2016) from Alta Floresta, southern Amazon. Brazil Molecular Phylogenetics and Evolution.

[ref-2] Boubli JP, Janiak MC, Porter LM, de la Torre S, Cortés-Ortiz L, Silva MNF, Rylands AB, Nash S, Bertuol F, Byrne H, Silva FE, Rohe F, Vries D, Beck RMD, Ruiz-Gartzia I, Kuderna LFK, Marques-Bonet T, Hrbek T, Farias IP, van Heteren AH, Roos C (2021). Ancient DNA of the pygmy marmoset type specimen *Cebuella pygmaea* (Spix, 1823) resolves a taxonomic conundrum. Zoological Research.

[ref-3] Boubli JP, Silva MNF, Rylands AB, Nash SD, Bertuol F, Nunes M, Mittermeier RA, Byrne H, Silva FE, Rohe F, Sampaio I, Schneider H, Farias IP, Hrbek T (2018). How many pygmy marmoset (*Cebuella* Gray, 1870) species are there? A taxonomic re-appraisal based on new molecular evidence. Molecular Phylogenetics and Evolution.

[ref-4] Bouckaert R, Heled J, Kühnert D, Vaughan T, Wu CH, Xie D, Suchard MA, Rambaut A, Drummond AJ (2014). BEAST 2: a software platform for Bayesian evolutionary analysis. PLOS Computational Biology.

[ref-5] Buckner JC, Lynch Alfaro JW, Rylands AB, Alfaro ME (2015). Biogeography of the marmosets and tamarins (Callitrichidae). Molecular Phylogenetics and Evolution.

[ref-6] Chifman J, Kubatko L (2014). Quartet inference from SNP data under the coalescent model. Bioinformatics.

[ref-7] Coimbra RTF, Winter S, Kumar V, Koepfli K-P, Gooley RM, Dobrynin P, Fennessy J, Janke A (2021). Whole-genome analysis of giraffe supports four distinct species. Current Biology.

[ref-8] Costa-Araújo R, Melo FR, Canale GR, Hernández-Rangel SM, Messias MR, Rossi RV, Silva FE, Silva MNF, Nash SD, Boubli JP, Farias IP, Hrbek T (2019). The Munduruku marmoset: a new monkey species from southern Amazonia. PeerJ.

[ref-9] Costa-Araújo R, Silva JS, Boubli JP, Rossi RV, Canale GR, Melo FR, Bertuol F, Silva FE, Silva DA, Nash SD, Sampaio I, Farias IP, Hrbek T (2021). An integrative analysis uncovers a new, pseudo-cryptic species of Amazonian marmoset (Primates: Callitrichidae: *Mico*) from the arc of deforestation. Scientific Reports.

[ref-10] de Queiroz K, Wilson RA (1999). The General Lineage Concept of species and the defining properties of the species category. Species: New Interdisciplinary Essays.

[ref-11] de Queiroz K (2007). Species concepts and species delimitation. Systematic Biology.

[ref-12] Eaton DAR (2014). PyRAD: assembly of de novo RADseq loci for phylogenetic analyses. Bioinformatics.

[ref-13] Fujita MK, Leaché AD, Burbrink FT, McGuire JA, Moritz CC (2012). Coalescent-based species delimitation in an integrative taxonomy. Trends in Ecology & Evolution.

[ref-14] Garbino GST (2015). How many marmoset (Primates: Cebidae: Callitrichinae) genera are there? A phylogenetic analysis based on multiple morphological systems. Cladistics.

[ref-15] Garbino GST, Martins-Junior AMG (2018). Phenotypic evolution in marmoset and tamarin monkeys (Cebidae, Callitrichinae) and a revised genus-level classification. Molecular Phylogenetics and Evolution.

[ref-16] Gauthier J, Mouden C, Suchan T, Alvarez N, Arrigo N, Riou C, Lemaitre C, Peterlongo P (2020). DiscoSnp-RAD: de novo detection of small variants for RAD-Seq population genomics. PeerJ.

[ref-17] Gregorin R, Vivo M (2013). Revalidation of *Saguinus ursula* Hoffmannsegg (Primates: Cebidae: Callitrichinae). Zootaxa.

[ref-18] Groves CP (2001). Primate taxonomy.

[ref-19] Grummer JA, Bryson RW, Reeder TW (2014). Species delimitation using Bayes Factors: simulations and application to the *Sceloporus scalaris* species group (Squamata: Phrynosomatidae). Systematic Biology.

[ref-20] Hanihara T, Natori M (1987). Preliminary analysis of numerical taxonomy of the genus *Saguinus* based on dental measurements. Primates.

[ref-21] Hershkovitz P (1968). Metachromism or the principle of evolutionary change in mammalian tegumentary colors. Evolution.

[ref-22] Hershkovitz P (1977). Living new world monkeys (Platyrrhini).

[ref-23] Heymann EW, Ravetta AL, Rohe F, Mittermeier RA (2021). *Saguinus mystax* ssp. *mystax*.

[ref-24] Hillis DM, Bull JJ (1993). An empirical test of bootstrapping as a method for assessing confidence in phylogenetic analysis. Systematic Biology.

[ref-25] International Commission on Zoological Nomenclature (1999). International code of zoological nomenclature.

[ref-26] Isaac NJ, Mallet J, Mace GM (2004). Taxonomic inflation: its influence on macroecology and conservation. Trends in Ecology and Evolution.

[ref-27] Johns AD (1985). Primate and forest exploitation at Tefé, Brazilian Amazonia. Primate Conservation.

[ref-28] Junier T, Zdobnov EM (2010). The Newick utilities: high-throughput phylogenetic tree processing in the UNIX shell. Bioinformatics.

[ref-29] Kanazawa E, Rosenberger AL (1988). Reduction index of the upper M2 in marmosets. Primates.

[ref-30] Kass RE, Raftery AE (1995). Bayes factors. Journal of the American Statistical Association.

[ref-31] Lanfear R, Frandsen PB, Wright AM, Senfeld T, Calcott B (2017). PartitionFinder 2: new methods for selecting partitioned models of evolution for molecular and morphological phylogenetic analyses. Molecular Biology and Evolution.

[ref-32] Leaché AD, Fujita MK, Minin VN, Bouckaert RR (2014). Species delimitation using genome-wide SNP data. Systematic Biology.

[ref-33] Malinsky M, Trucchi E, Lawson DJ, Falush D (2018). RADpainter and fineRADstructure: population inference from RADseq data. Molecular Biology and Evolution.

[ref-34] Mallet J, Isaac NJB, Mace GM (2005). Response to Harris and Froufe, and Knapp et al.: taxonomic inflation. Trends in Ecology and Evolution.

[ref-35] Mirarab S (2019). Species tree estimation using ASTRAL: practical considerations. arXiv preprint.

[ref-36] Mittermeier RA, Schwarz M, Ayres JM (1992). A new species of marmoset, genus *Callithrix* Erxleben 1777 (Callitrichidae, Primates), from the Rio Maués region, state of Amazonas, Central Brazilian Amazonia. Goeldiana Zoologia.

[ref-37] Moore AJ, Cheverud JM (1992). Systematics of the *Saguinus oedipus* group of the bare-face tamarins: evidence from facial morphology. American Journal of Physical Anthropology.

[ref-38] Munsell Color (2000). Munsell soil color charts.

[ref-39] Nater A, Mattle-Greminger MP, Nurcahyo A, Nowak MG, Manuel M, Desai T, Groves C, Pybus M, Sonay TB, Roos C, Lameira AR, Wich SA, Askew J, Davila-Ross M, Fredriksson G, Valles G, Casals F, Prado-Martinez J, Goossens B, Verschoor EJ, Warren KS, Singleton I, Marques DA, Pamungkas J, Perwitasari-Farajallah D, Rianti P, Tuuga A, Gut IG, Gut M, Orozco-terWengel P, van Schaik CP, Bertranpetit J, Anisimova M, Scally A, Marques-Bonet T, Meijaard E, Krützen M (2017). Morphometric, behavioral, and genomic evidence for a new orangutan species. Current Biology.

[ref-40] Natori M, Hanihara T (1992). Variations in dental measurements between *Saguinus* species and their systematic relationships. Folia Primatologica.

[ref-41] Nute M, Chou J, Molloy EK, Warnow T (2018). The performance of coalescent-based species tree estimation methods under models of missing data. BMC Genomics.

[ref-42] Peterson BK, Weber JN, Kay EH, Fisher HS, Hoekstra HE (2012). Double digest RADseq: an inexpensive method for de novo SNP discovery and genotyping in model and non-model species. PLOS ONE.

[ref-44] Ravetta A, Rohe F (2020a). *Saguinus mystax* ssp. *pileatus*.

[ref-45] Ravetta A, Rohe F (2020b). *Saguinus mystax* ssp. *pluto*.

[ref-46] Rohe F, Silva JS, Sampaio R, Rylands AB (2009). A new subspecies of *Saguinus fuscicollis* (Primates, Callitrichidae). International Journal of Primatology.

[ref-47] Rylands AB (1993). The bare-face tamarins *Saguinus oedipus oedipus* and *Saguinus oedipus geoffroyi*: subspecies or species?. Neotropical Primates.

[ref-48] Rylands AB, Coimbra-Filho AF, Mittermeier RA, Rylands AB (1993). Systematics, geographic distributions, and some notes on the conservation status of the Callitrichidae. Marmosets and Tamarins: Systematics, Behaviour and Ecology.

[ref-49] Rylands AB, Heymann EW, Lynch Alfaro JW, Buckner JC, Roos C, Matauschek C, Boubli JP, Sampaio R, Mittermeier RA (2016). Taxonomic review of the New World tamarins (Primates: Callitrichidae). Zoological Journal of the Linnean Society.

[ref-50] Rylands AB, Mittermeier RA, Garber PA, Estrada A, Bicca-Marques JC, Heymann EW, Strier KB (2009). The diversity of the New World primates: an annotated taxonomy. South American Primates: Comparative Perspectives in the Study of Behavior, Ecology, and Conservation.

[ref-52] Rylands AB, Schneider H, Langguth A, Mittermeier RA, Groves CP, Rodríguez-Luna E (2000). An assessment of the diversity of New World primates. Neotropical Primates.

[ref-53] Sambrook J, Russell RW (2001). Molecular cloning: a laboratory manual.

[ref-54] Sampaio R, Rohe F, Pinho GM, Silva JS, Farias IP, Rylands AB (2015). Re-description and assessment of the taxonomic status of *Saguinus fuscicollis cruzlimai* Hershkovitz, 1966 (Primates, Callitrichinae). Primates.

[ref-55] Sampaio R, Rohe F, Rylands AB (2018). Diversity of primates and other mammals in the middle Purus basin in the Brazilian Amazon. Mammalia.

[ref-57] Silva FE, Costa-Araújo R, Boubli JP, Santana MI, Franco CLB, Bertuol F, Nunes H, Silva-Júnior JS, Farias I, Hrbek T (2018). In search of a meaningful classification for Amazonian marmosets: should dwarf marmosets be considered *Mico* congenerics?. Zoologica Scripta.

[ref-58] Silva JS, Noronha MA (1998). On a new species of bare-eared marmoset, genus *Callithrix* Erxleben, 1777, from central Amazonia, Brazil (Primates: Callitrichidae). Goeldiana Zoologia.

[ref-59] Skinner C (1991). Justification for reclassifying Geoffroy’s tamarin from *Saguinus oedipus geoffroyi* to *Saguinus geoffroyi*. Primate Report.

[ref-60] Stamatakis A (2014). RAxML version 8: a tool for phylogenetic analysis and post-analysis of large phylogenies. Bioinformatics.

[ref-61] Swofford DL (2002). PAUP*. Phylogenetic analysis using parsimony (*and Other Methods). http://paup.phylosolutions.com/.

[ref-62] Vallinoto M, Araripe J, Rego PS, Tagliaro CH, Sampaio I, Schneider H (2006). Tocantins River as an effective barrier to gene flow in *Saguinus niger* populations. Genetics and Molecular Biology.

[ref-63] van Roosmalen MGM, van Roosmalen T (2003). The description of a new marmoset genus, *Callibella* (Callitrichinae, Primates), including its molecular phylogenetic status. Neotropical Primates.

[ref-64] Vivo MD (1991). Taxonomia de Callithrix Erxleben, 1777 (Callitrichidae, Primates).

[ref-65] Zhang C, Rabiee M, Sayyari E, Mirarab S (2018). ASTRAL-III: polynomial time species tree reconstruction from partially resolved gene trees. BMC Bioinformatics.

[ref-66] Zhang C, Sayyari E, Mirarab S, Meidanis J, Nakhleh L (2017). ASTRAL-III: increased scalability and impacts of contracting low support branches. Lecture Notes in Computer Science.

