# Supplementary figures and images for: Taxonomic review of Saguinus mystax (Spix, 1823) (Primates, Callitrichidae), and description of a new species
Source: PeerJ. 2023 Jan 11;11:e14526. doi: 10.7717/peerj.14526 (PMC9840391; doi:10.7717/peerj.14526)

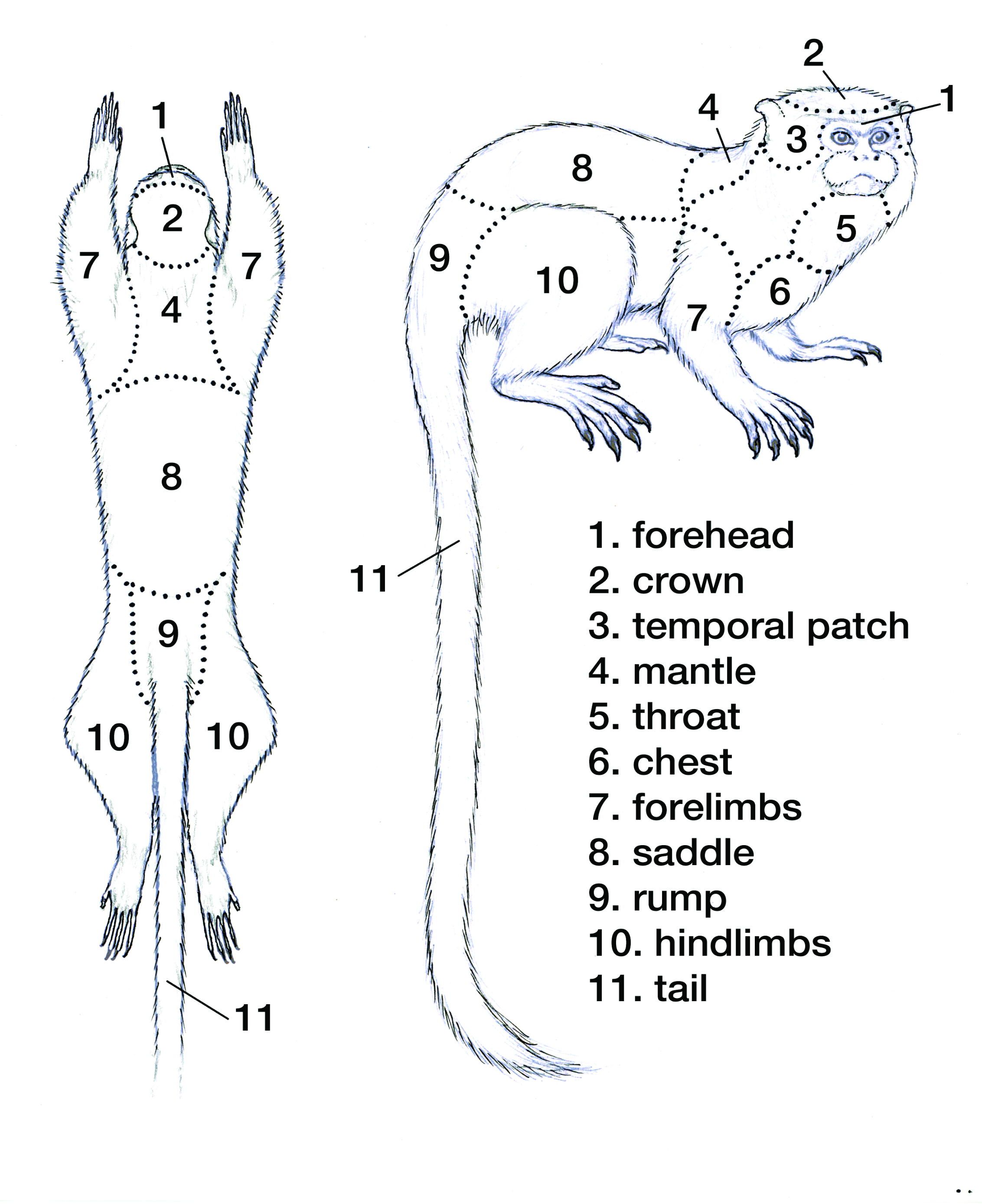

Supplement: Supplemental Information 1 [file peerj-11-14526-s001.png]

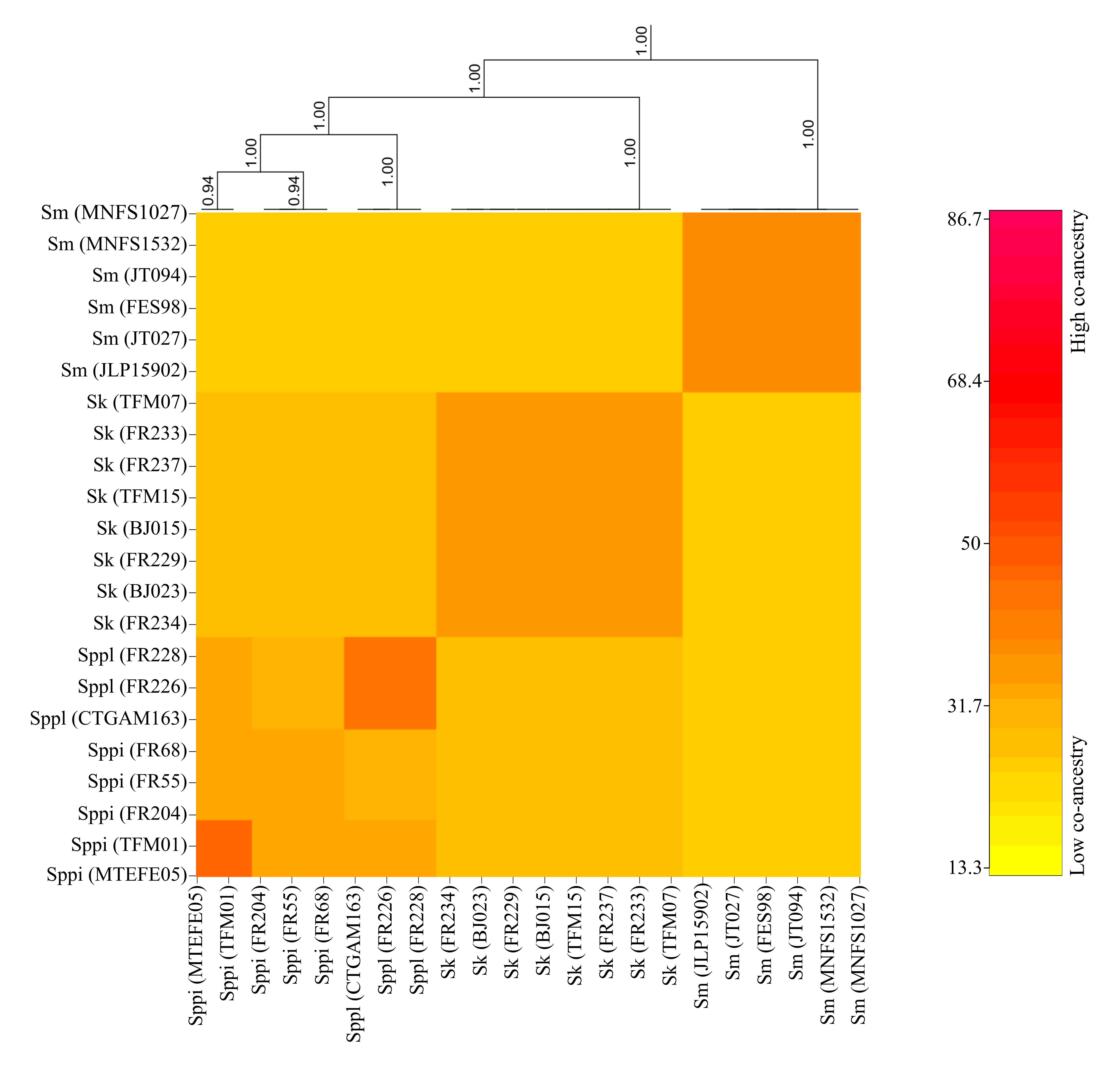

Supplement: Supplemental Information 2 — Individuals within the same species/subspecies share more coancestry with each other than other species/subspecies, indicated by colors. Sm = Saguinus mystax, Sk = Saguinus kulina, Sppl = Saguinus pileatus pluto, Sppi = Saguinus pileatus pileatus. [file peerj-11-14526-s002.png]
