# Supplementary material for: Taxonomic review of Saguinus mystax (Spix, 1823) (Primates, Callitrichidae), and description of a new species
Source: PeerJ. 2023 Jan 11;11:e14526. doi: 10.7717/peerj.14526 (PMC9840391; doi:10.7717/peerj.14526)
Supplement: Supplemental Information 3 [file peerj-11-14526-s003.docx]

Supplementary Table 1. Specimens, field number/collection numbers, locality, and geographic coordinates.

| **Taxon** | **Field number (Specimen code)** | **Locality** | **Geographic coordinates** | |
| --- | --- | --- | --- | --- |
|  |  |  | **Lat.** | **Long.** |
| *S*. *m*. *pileatus* | AAM9 | Right bank of Solimões River, Amazonas, Brazil | - 3.855778 | -63.89725 |
| *S*. *m*. *pileatus* | FR55 (INPA5684) | Right bank of Juruá River, Amazonas, Brazil | -5.4392 | -67.2734 |
| *S*. *m*. *pileatus* | FR68 (INPA5687) | Right bank of Moaco River, Amazonas, Brazil | -7.627778 | -69.113056 |
| *S*. *m*. *pileatus* | FR204 | Paissé River, left bank of Purus River, Amazonas | -6.50347 | -64.56162 |
| *S*. *m*. *pileatus* | MTEFE05 (IDSM03680) | Right bank of Tefé River, Amazonas, Brazil | -3.355282 | -64.731128 |
| *S*. *m*. *pileatus* | TFM01 (IDSM03384) | Right bank of Tefé River, Amazonas, Brazil | - 3.455758 | - 64.76483 |
| *S. m*. *pluto* | CTGAM163 | Left bank of Purus River, Amazonas, Brazil | -4.980583 | -62.977694 |
| *S. m*. *pluto* | FR226 | Right bank of Coari River, Amazonas, Brazil | -4.172417 | -63.160194 |
| *S. m*. *pluto* | FR228 | Right bank of Coari River, Amazonas, Brazil | -4.172417 | -63.160194 |
| *S. m*. *mystax* | FES98 (IDSM03681) | Right bank of Moa River, Acre, Brazil | -7.451228 | -73.678022 |
| *S. m*. *mystax* | FR210 | Right bank of Moa River, Acre, Brazil | -7.507056 | -73.706833 |
| *S. m*. *mystax* | JLP15902 | Left bank of Juruá River, Amazonas, Brazil | -6.466667 | -68.766667 |
| *S. m*. *mystax* | JT27 (IDSM00045) | Right bank of Jutaí River, Amazonas, Brazil | -3.266891 | -67.324052 |
| *S. m*. *mystax* | JT52 (IDSM00067) | Left bank of Jutaí River, Amazonas, Brazil | -3.178177 | -67.391922 |
| *S. m*. *mystax* | JT94 (IDSM00778) | Right bank of Jutaí River, Amazonas, Brazil | -3.720148 | -67.445046 |
| *S. m*. *mystax* | MNFS1027 | Left bank of Juruá River, Acre, Brazil | -8.6 | -72.85 |
| *S. m*. *mystax* | MNFS1532 | Left bank of Juruá River, Acre, Brazil | -8.366667 | -72.816667 |
| *Saguinus* sp. | BJ015 (IDSM03594) | Right bank of Juruá River, Amazonas, Brazil | -3.732359 | -66.09523 |
| *Saguinus* sp. | BJ023 (IDSM03602) | Right bank of Juruá River, Amazonas, Brazil | -4.35 | -73.15 |
| *Saguinus* sp. | FR229 | Right bank of Juruá River, Amazonas, Brazil | -4.7945 | -66.616742 |
| *Saguinus* sp. | FR233 | Right bank of Juruá River, Amazonas, Brazil | - 4.7945 | -66.616742 |
| *Saguinus* sp. | FR234 | Right bank of Juruá River, Amazonas, Brazil | - 4.816681 | -66.583722 |
| *Saguinus* sp. | FR237 | Right bank of Juruá River, Amazonas, Brazil | -4.750239 | -66.600872 |
| *Saguinus* sp. | TFM07 (IDSM03652) | Left bank of Tefé River, Amazonas, Brazil | - 3.294625 | -64.795972 |
| *Saguinus* sp. | TFM15 (IDSM03659) | Left bank of Tefé River, Amazonas, Brazil | -3.288844 | -64.773347 |
